# 2214. Retrospective Analysis of the Implementation of a Novel Discharge Reconciliation service in OPAT by Pharmacists (RAINDROP)

**DOI:** 10.1093/ofid/ofad500.1836

**Published:** 2023-11-27

**Authors:** Brady Caverzagie, Jennifer Ross

**Affiliations:** M Health Fairview University of Minnesota Medical Center, Minneapolis, Minnesota; M Health Fairview - University of Minnesota Medical Center, Minneapolis, MN

## Abstract

**Background:**

Outpatient parenteral antimicrobial therapy (OPAT) has become a standard mechanism for delivery of antimicrobial regimens over a prolonged period in a non-acute care setting. Adverse drug reactions, vascular access device complications, and unscheduled healthcare use have been attributable to OPAT. Existing literature suggests OPAT errors at the time of hospital discharge are high, around 30-40%. The purpose of this study is to evaluate the impact of a novel OPAT reconciliation service on pharmacist interventions on OPAT discharge plans.

**Methods:**

Adult OPAT patients who discharged from an academic medical center between 6/2020 and 6/2023 on parenteral antimicrobial(s) with placement of a pharmacist OPAT note were included in this retrospective pre/post implementation cohort study. Patients with cystic fibrosis were excluded. Data on patient characteristics, antibiotic regimen, labs, and any changes to the discharge OPAT plan were collected from the electronic health record. Categorical values and outcomes were reported as counts and rates.

**Results:**

The implementation of a new OPAT plan reconciliation process for infectious diseases pharmacists was implemented on January 18th, 2023, and is outlined in Figure 1. Target enrollment will be approximately 640 patients split evenly between two cohorts. An interim analysis was performed on 553 patients, 320 patients in the pre-implementation cohort and 233 in the post implementation cohort. There was a total of 53 pharmacist interventions observed during the pre-implementation period and 61 interventions in the post-implementation period, yielding an intervention rate of 0.17 (6.04 patients/intervention) and 0.26 (3.82 patients/intervention), respectively. Common interventions included changes to lab and therapeutic drug monitoring, dose, agent chosen with culture follow-up, and changes to the infusion method (continuous vs. intermittent infusion). A breakdown of the interventions observed in the two cohorts is shown in Table 1.

**Figure 1:**
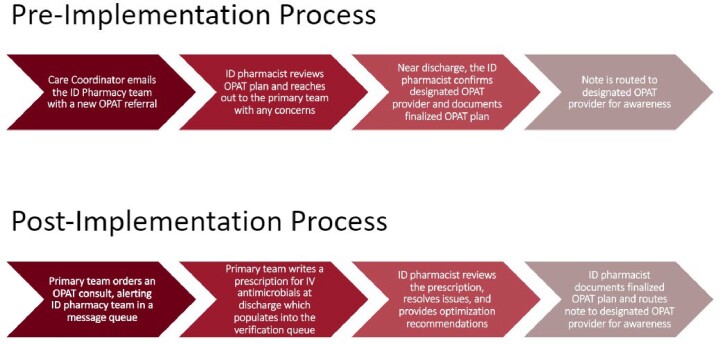
Pre and Post Implementation OPAT Plan Reconciliation Process for Infectious Disease Pharmacists

**Table 1:**
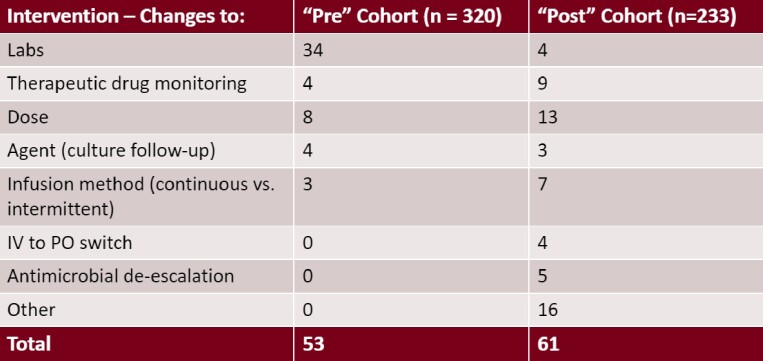
Pre and Post Implementation Infectious Disease Pharmacist Intervention Type Breakdown

**Conclusion:**

During our interim analysis, we observed an increased rate of pharmacist interventions after the start of a new reconciliation service for OPAT discharges.

**Disclosures:**

**All Authors**: No reported disclosures

